# Factors associated with cognitive function in older adults in Mexico

**DOI:** 10.3402/gha.v9.30747

**Published:** 2016-03-30

**Authors:** Jenny Miu, Joel Negin, Aarón Salinas-Rodriguez, Betty Manrique-Espinoza, Ana Luisa Sosa-Ortiz, Robert Cumming, Paul Kowal

**Affiliations:** 1 School of Public Health, Sydney Medical School, University of Sydney, Sydney, Australia; 2 Center for Evaluation Research and Surveys, National Institute of Public Health, Cuernavaca, Morelos, Mexico; 3 Laboratory of Dementias, National Institute of Neurology and Neurosurgery, Mexico DF, Mexico; 4 Research Centre for Generational Health and Ageing, Faculty of Health, University of Newcastle, Newcastle, Australia; 5 World Health Organization Study on global AGEing and adult health (SAGE), Geneva, Switzerland

**Keywords:** ageing, cognition, epidemiological studies, Mexico

## Abstract

**Background:**

As populations age, cognitive decline and dementia pose significant burdens for societies and health care systems, including low- and middle-income countries such as Mexico. Minor age-related declines in cognitive function appear to represent a stable but heterogeneous phase in the continuum between normal cognitive ageing and dementia. Loss of cognitive function has impacts at societal and individual levels and understanding the risk factors can help provide a framework for health policies and interventions to target at-risk groups.

**Design:**

A cohort of older Mexican adults (50+) from the World Health Organization's Study on global AGEing and adult health (WHO SAGE) was used to examine cognitive function, including a total of 2315 respondents, with 325 respondents aged 80 years and older. Cognition was objectively evaluated using verbal recall, verbal fluency, forward digit span and backward digit span, with differences in an overall cognitive score assessed against sociodemographic variables, and associated factors using linear regression.

**Results:**

The most significant predictors of poorer cognitive function were found to be older age (β=−13.88), rural living (β=−2.25), low income (β=−8.28), self-reported severe or extreme memory difficulties (β=−6.62), and difficulty with two or more activities of daily living (β=−2.02).

**Conclusions:**

These findings can inform public health initiatives to address cognitive impairment in ageing populations in Mexico and other middle-income countries.

## Introduction

Dementia and cognitive impairment are significant sources of morbidity in ageing populations around the world, including middle-income countries such as Mexico. Mexico is the fifth largest country in the Americas with a population over 120 million, and a life expectancy of 80 years for women and 75 for men; almost 20% are expected to be aged 60 and older by 2030 (1). Overall, developing countries currently carry 58% of the world's dementia burden and this is projected to rise to 71% by 2050 (2). Mexico has a high incidence rate of dementia (30.4 per 1,000 person-years) in those aged 65 and older, when compared to other countries in the Latin American region (3), and is estimated to affect 6–8% of those aged 60 years (4, 5). Dementia, which includes Alzheimer's disease, Lewy body and frontotemporal dementias, and vascular dementia, results from a number of pathological processes and is preceded by changes in cognition that differ from the normal ageing process. Mild changes in cognition can manifest as delays in memory and impairments in complex activities of daily living (ADLs) and instrumental activities of daily living (IADLs) (6, 7). More significant cognitive impairment would be characterised by considerable disability and neuropsychiatric symptoms such as depression and anxiety (8), often resulting in high caregiving needs (9). In Mexico, estimates are 3–29% for mild cognitive impairment for those aged over 60 years (10, 11), compared to a general estimate of 10–20% in older adult populations (6).

The evidence on risk factors for dementia is growing, with the strongest evidence currently available for advancing age, low education levels in early life, tobacco use, diabetes, and midlife hypertension (12–14), and may be explained in part by the effect of education on cognitive reserve (15) or changes in brain structure and function due to vascular disease (16). In Mexican populations, reported risk factors for mild impairments in cognition include older age, sex, education, depression, heart disease, and diabetes (10, 11). As ageing populations are growing worldwide, groups at highest risk for cognitive decline, particularly those from low- and middle-income countries, can be targeted as part of wider efforts to reduce the prevalence of dementia and its burden on society and healthcare systems. This paper examined risk factors for cognitive impairment amongst a sample of adults aged 50 years and older in a population-based study in Mexico.

## Methods

### Population and sample

The sample of respondents aged 50+ years from the World Health Organization's Study on global AGEing and adult health (WHO SAGE) Mexico Wave 1 (2009–2010) was used for this study. SAGE is a longitudinal study of health and well-being in adults from six low- and middle-income countries, including Mexico. Stratified multistage clustered sampling (1) was used to generate a final sample of 2,756 respondents in Mexico. The study design included samples of follow-up respondents from SAGE Wave 0, a baseline cohort created during the 2002–2004 World Health Survey, and new respondents. Proxy respondents were identified for respondents who were unable to provide reliable responses or due to poor health. Face-to-face interviews were conducted in Mexico between 2009 and 2010. Cross-sectional data from SAGE Wave 1 were used in this study. More information on WHO SAGE is available elsewhere (17). SAGE was approved by the Ethics Review Committee of the World Health Organization and by the Ethics Committee of the National Institute of Public Health in Cuernavaca, Mexico.

### Measures

SAGE evaluates self-reported perceptions of health, well-being, and cognition, and also assesses these using more objective measures, including a battery of cognitive tests (immediate and delayed verbal recall, VR; verbal fluency, VF; forward digit span, FDS; and backward digit span, BDS), and a 4-metre (4-m) timed walk at both normal and rapid pace.

Verbal memory was tested using verbal recall, consisting of three repetitions of a 10-word list for immediate recall, and then assessing the recall of these words after a 10-min delay. The verbal fluency test consisted of naming as many animals as possible in 1 min, to assess attention, semantic memory, and executive functions. Digit span was used to assess memory capacity and executive function, using both forward and backward digit recall tests (18). An overall composite cognitive score, used to characterise cognitive function, was calculated by standardising and summing the correct responses from immediate and delayed VR, VF, FDS, and BDS, and rescaling these to a value between 0 and 100, where 0 is the worst and 100 is the best score for cognition (19).

Covariates included a number of sociodemographic variables such as age, sex, urban/rural locality of residence and asset-based income quintiles. Age was recategorised into four age groups: 50–59, 60–69, 70–79, and 80+. Other predictors included: self-report of difficulties with memory (‘Overall in the last 30 days, how much difficulty did you have with concentrating or remembering things?’), difficulties with ADLs (including questions on eating, walking, climbing stairs, concentrating, bathing, getting dressed) and IADLs (including questions on household care, daily work, using public transport), timed normal gait speed over a 4 m distance (slow gait was considered to be <1 m/s (20) with gait speeds <0.13 m/s and >4 m/s excluded). Prevalence of stroke and hypertension was based on self-report using a form of the question: ‘Have you ever been told by a health professional that you have [*disease/condition*]?’. Depression was measured using an algorithm based on diagnosis and symptoms (including questions on medication/therapy, sadness, loss of interest, low energy) and based on the World Mental Health Survey's version of the Composite International Diagnostic Interview (21). Use of tobacco was based on the question: ‘Do you currently use (smoke, sniff or chew) any tobacco products such as cigarettes, cigars, pipes, chewing tobacco or snuff?’. Tobacco use for this analysis included daily and non-daily use. Alcohol use was based on the question: ‘Have you consumed alcohol in the last 30 days?’.

### Statistical analysis

Data were analysed in STATA (MP13). Post-stratification analysis weights were applied. Bivariate associations between predictors and the outcome variable (overall cognitive score) were assessed using linear regression. Reference groups were: 50–59 age group, male gender, urban locality, lowest income percentile, no self-reported difficulty with memory, no difficulty with any of the six ADLs, no difficulty with IADLs, slow gait speed (<1 m/s), no depression, no stroke, no hypertension, no tobacco use, and no alcohol use. Variables associated with cognition at *p*<0.1 were then included in a multiple linear regression analysis and reduced stepwise until only factors with *p*<0.05 remained.

## Results

Of the 2,315 respondents, 19% were aged 50–59, 41% were aged 60–69, 27% were aged 70–79, and 14% were 80+ years old (Table 1, unweighted data). Men made up 40% of respondents and 73% of respondents lived in urban areas. A large proportion of people reported having memory difficulties in the past 30 days (53%) and difficulties in ADLs (45%); only 16% reported difficulties with IADLs. Respondents predominantly walked at a gait slower than 1 m/s (85%). Thirteen percent of older adults had a history of depression, 5% reported having had a stroke, and 39% reported hypertension. Fifty-two percent reported current tobacco use and 28% had consumed alcohol in the past 30 days.

**Table 1:** Mean overall cognition score, by selected sociodemographic characteristics and associated covariates and risks

	Number (%)^a^	Overall cognition score^b^	Significance^c^
Mean		52.4	
**Sociodemographic characteristics**			
Age			
50–59	434 (19)	56.4	
60–69	937 (41)	53.4	***
70–79	619 (27)	47.1	***
>80	325 (14)	37.3	***
Sex			
Men	911 (40)	52.8	
Women	1,395 (60)	52.2	ns
Locality			
Urban	1,689 (73)	53.7	
Rural	626 (27)	47.7	***
Income (quintiles)			
Lowest	497 (22)	45.1	
Low	489 (21)	48.7	***
Moderate	420 (18)	52.7	***
High	472 (20)	54.1	***
Highest	433 (19)	59.1	***
**Covariates**			
Self-reported memory difficulty			
None	1,038 (47)	54.6	
Mild/moderate	1,097 (50)	51.8	***
Severe/extreme	76 (3)	44.2	***
Difficulty with ADLs			
0	1,215 (55)	54.6	
1	303 (14)	53.6	ns
2+	693 (31)	47.9	***
Difficulty with IADLs			
0	1,871 (85)	53.7	
1	122 (6)	51.4	ns
2+	216 (10)	43.8	***
Gait speed			
Slow (<1 m/s)	1,586 (85)	52.6	
Fast	276 (15)	56.0	***
Depression			
No	1,913 (87)	52.9	
Yes	295 (13)	54.0	ns
Stroke			
No	2,103 (95)	53.1	
Yes	106 (5)	51.1	ns
Hypertension			
No	1,374 (62)	53.5	
Yes	834 (39)	52.1	*
Tobacco use			
No	404 (48)	52.4	
Yes	437 (52)	57.2	***
Alcohol use			
No	760 (72)	52.2	
Yes	298 (28)	57.6	***

a Data are unweighted.

b Data are weighted, where 0 is worst and 100 is best cognition.

c Reference groups: aged 50–59, male sex, urban locality, lowest income quintile, no memory difficulty, no ADL difficulty, no IADL difficulty, slow gait speed (<1 ms), no depression, no stroke, no hypertension, no tobacco use, no alcohol use in past 30 days

*** *p*<0.001

** *p*<0.01

* *p*<0.05; ns=not significant (simple linear regression), ADLs=activities of daily living, IADLs=instrumental activities of daily living.

In the bivariate analysis, significant negative associations were found between lower cognitive score (poorer cognitive function) and higher age (*p*<0.001), rural living (*p*<0.001), increasing difficulty with memory (*p*<0.001), increasing difficulty with ADLs (*p*<0.001), and IADLs (*p*<0.001), and those diagnosed with hypertension (*p=*0.013) (Table 1, weighted data). Positive associations were observed for income percentile (*p*<0.001), faster gait speed (*p*<0.001), current tobacco use (*p*<0.001), and alcohol use in the past 30 days (*p*<0.001) (Table 1, weighted data). Sex, depression, and stroke were not significantly associated with the calculated composite cognitive score.

Multiple linear regression analyses revealed that, after adjusting for confounding factors, poorer global cognitive functioning was significantly associated with age (>80, β=−13.88, *p*<0.001), locality (rural living, β=−2.25, *p*<0.01), income quintile (lowest, β=−8.28, *p*<0.001), difficulties with memory (severe/extreme, β=−6.62, *p*<0.01), and ADLs (2+, β=−2.02, *p*<0.05) as well as no reported alcohol consumption (β=−2.22, *p*<0.001) (Table 2, weighted data), suggesting that both modifiable and non-modifiable factors contribute to cognitive function.

**Table 2:** Stepwise multiple linear regression analysis of factors associated with overall cognitive score

	β^a^	95% CI	Significance^b^
**Sociodemographic characteristics**			
Age			
50–59			
60–69	−1.69	−3.07 to −0.31	*
70–79	−6.66	−8.49 to −4.82	***
>80	−13.88	−16.50 to −11.25	***
Locality			
Urban			
Rural	−2.25	−3.70 to −0.80	**
Income (quintiles)			
Lowest			
Low	−1.00	−3.07 to 1.05	ns
Moderate	3.53	1.32 to 5.73	**
High	4.78	2.60 to 6.97	***
Highest	8.28	6.18 to 10.37	***
**Covariates**			
Self-reported memory difficulty			
None			
Mild/moderate	−1.38	−2.53 to −0.22	*
Severe/extreme	−6.62	−10.97 to −2.28	**
ADLs			
0			
1	0.22	−1.32 to 1.76	ns
2+	−2.02	−3.66 to −0.38	*
Alcohol use			
No			
Yes	2.22	1.04 to 3.40	***

a Data are weighted.

b Reference groups: aged 50–59, urban locality, lowest income quintile, no memory difficulty, no ADL difficulty, no alcohol use in past 30 days

*** *p*<0.001

** *p*<0.01

* *p*<0.05; ns=not significant (multiple regression), ADLs=activities of daily living.

## Discussion

Impaired cognitive function is becoming an important source of morbidity as populations age, with cognitive changes and dementia representing significant burdens in Mexico (3–5, 10, 11). Understanding the population at greatest risk can lead to targeted interventions and more effective use of limited health resources in Mexico and similar developing countries (14). In this study, lower cognitive functioning was strongly associated with advancing age, rural living and lower income, as well as with subjective memory complaints and ADL limitations.

These findings compare with other SAGE publications examining cognition in Ghana, Tanzania, India (18), South Africa (22), and China (23), where poorer cognitive function was observed in those of advancing age, rural living, lower education, and wealth levels, and poorer subjective health status, including deterioration in ADLs and slower gait. Subjective losses in memory and daily functioning and its association with poorer cognition have been described as features of mild cognitive impairment (6, 7), and testing these along with gait speed (24) may aid the early detection of those at high risk of dementia and provide a point for successful interventions.

Comorbidities, such as hypertension, also indicated a higher risk for poorer cognitive functioning in this study, but this was not observed for a history of stroke as shown in previous studies in the Mexican population (10, 11). Although evidence suggests a role for vascular disease in cognitive changes as a result of changes in brain structure and function (16), epidemiological evidence in studies involving Mexican and Mexican–American populations is inconsistent in regards to the precise relationship between cognitive function and hypertension and stroke. Hypertension may be a stronger predictor of dementia (10) rather than cognitive impairment (11, 25) and this study did not include a measure of hypertension in midlife, the factor with, perhaps, the strongest evidence (26). There also is a lack of causal evidence between cognition and stroke, as both have been described on the causal pathway in Mexican–American populations (25, 27). Similarly, depression may (28), or may not (25) be associated with cognitive changes.

Modifiable factors such as tobacco and alcohol use did not have a negative association with cognitive function, perhaps because the duration and quantity of use were not quantified in these regressions. Approximately 33% of tobacco users were not daily smokers (data not shown). While tobacco is generally associated with poorer cognitive functioning (13, 29), as it is a known risk factor for cardiovascular and cerebrovascular disease (30), some studies have not shown an association (31). These differences are attributed to survivorship bias in large epidemiologic studies (32). Similarly, while alcohol in moderation has been shown to be protective (29, 33) other studies have shown no association with cognitive decline (13). This may reflect the positive and negative effects of alcohol on the cardiovascular system (33), resulting in a relationship described as U-shaped. Alcohol consumption also was not quantified for this analysis but of the respondents who reported use in the past 30 days, 54% had consumed less than one standard drink in the past month (data not shown).

This study describes for the first time associations between various demographic and modifiable risk factors and global measures of cognitive function in a representative sample of the older adult Mexican population, building upon a previous study examining incidence rates of dementia and cognitive impairment (10). Strengths of this study include the large-scale cross-sectional data, including the collection of objective measurements, with the potential to follow up on individual data with future survey waves. Comparisons also can be made across a number of low- and middle-income countries that are participating in WHO SAGE. Weaknesses include the lack of causality, the use of subjective rather than objective measures including self-report on some disease conditions, missing data particularly on self-reporting which can bias these findings, and the exclusion of dementia data. These findings can inform public health initiatives in Mexico, for example, the current Over 70s Allowance, the 70+ Program, or Prospera an intervention for those living in poverty (1), to address wider issues involving ageing populations by increasing education and supporting memory (34), or improving vascular health, to reduce the burden of cognitive impairment.

## Authors’ contributions

JM designed the study, carried out the analyses, and wrote the paper. JN and PK designed the study, supervised the analyses, and wrote the paper. AS, BM, ALS, and RC reviewed and modified with their contributions to the original manuscript. All authors have read and approved of the final version of the manuscript.
